# Community and health system intervention to reduce disrespect and abuse during childbirth in Tanga Region, Tanzania: A comparative before-and-after study

**DOI:** 10.1371/journal.pmed.1002341

**Published:** 2017-07-11

**Authors:** Stephanie A. Kujawski, Lynn P. Freedman, Kate Ramsey, Godfrey Mbaruku, Selemani Mbuyita, Wema Moyo, Margaret E. Kruk

**Affiliations:** 1 Department of Epidemiology, Mailman School of Public Health, Columbia University, New York, New York, United States of America; 2 Department of Population and Family Health, Mailman School of Public Health, Columbia University, New York, New York, United States of America; 3 Ifakara Health Institute, Dar es Salaam, Tanzania; 4 Department of Global Health and Population, Harvard T.H. Chan School of Public Health, Boston, Massachusetts, United States of America; Stellenbosch University, SOUTH AFRICA

## Abstract

**Background:**

Abusive treatment of women during childbirth has been documented in low-resource countries and is a deterrent to facility utilization for delivery. Evidence for interventions to address women’s poor experience is scant. We assessed a participatory community and health system intervention to reduce the prevalence of disrespect and abuse during childbirth in Tanzania.

**Methods and findings:**

We used a comparative before-and-after evaluation design to test the combined intervention to reduce disrespect and abuse. Two hospitals in Tanga Region, Tanzania were included in the study, 1 randomly assigned to receive the intervention. Women who delivered at the study facilities were eligible to participate and were recruited upon discharge. Surveys were conducted at baseline (December 2011 through May 2012) and after the intervention (March through September 2015). The intervention consisted of a client service charter and a facility-based, quality-improvement process aimed to redefine norms and practices for respectful maternity care. The primary outcome was any self-reported experiences of disrespect and abuse during childbirth. We used multivariable logistic regression to estimate a difference-in-difference model. At baseline, 2,085 women at the 2 study hospitals who had been discharged from the maternity ward after delivery were invited to participate in the survey. Of these, 1,388 (66.57%) agreed to participate. At endline, 1,680 women participated in the survey (72.29% of those approached). The intervention was associated with a 66% reduced odds of a woman experiencing disrespect and abuse during childbirth (odds ratio [OR]: 0.34, 95% CI: 0.21–0.58, *p* < 0.0001). The biggest reductions were for physical abuse (OR: 0.22, 95% CI: 0.05–0.97, *p* = 0.045) and neglect (OR: 0.36, 95% CI: 0.19–0.71, *p* = 0.003). The study involved only 2 hospitals in Tanzania and is thus a proof-of-concept study. Future, larger-scale research should be undertaken to evaluate the applicability of this approach to other settings.

**Conclusions:**

After implementation of the combined intervention, the likelihood of women’s reports of disrespectful treatment during childbirth was substantially reduced. These results were observed nearly 1 year after the end of the project’s facilitation of implementation, indicating the potential for sustainability. The results indicate that a participatory community and health system intervention designed to tackle disrespect and abuse by changing the norms and standards of care is a potential strategy to improve the treatment of women during childbirth at health facilities. The trial is registered on the ISRCTN Registry, ISRCTN 48258486.

**Trial registration:**

ISRCTN Registry, ISRCTN 48258486

## Abstract 2

In a comparative before-and-after study, Stephanie Kujawski and colleagues examine whether the risk of women experiencing any disrespect or abuse during childbirth is reduced following implementation of a community and health system intervention in Tanga Region, Tanzania.

## Author summary

**Why was the study done?:**

- There is growing evidence of the mistreatment of women during childbirth at health facilities, particularly in low-resource settings.
- Few studies have designed and evaluated interventions to directly address disrespect and abuse.

**What did the researchers do and find?:**

- We performed a comparative before-and-after evaluation of an intervention to address disrespect and abuse during childbirth.
- This study used a participatory community and health system intervention in 2 hospitals in Tanga Region, Tanzania.
- After nearly 1 year, the intervention was associated with a 66% reduced odds of a woman experiencing any disrespect or abuse during childbirth, with the biggest reductions observed for physical abuse and neglect.

**What do these findings mean?:**

- This study suggests that supporting frontline community- and facility-based actors to identify and act on the symptoms and causes of disrespect and abuse in their own settings may be associated with a reduction of disrespectful treatment.
- The results should be interpreted as a proof-of-concept study, as the intervention was tested in only 2 facilities.

## Introduction

Maternal health in the Millennium Development Goal (MDG) era (2000 through 2015) was dominated by a focus on increasing skilled birth attendance, typically through facility delivery, as a means to reducing maternal mortality [1]. Countries with high maternal mortality ratios (MMR) worked to remove barriers to delivery in health facilities by eliminating user fees, providing conditional cash transfers, improving transport, and scaling up emergency obstetric care [2,3]. In sub-Saharan Africa, the MMR dropped by 45% between 1990 and 2015, which was short of the 75% MDG target, and the region still accounts for 66% of all maternal deaths as of 2015 [4].

As the MDG era came to a close, new evidence called into question the prevailing strategy that focused so narrowly on increasing intervention coverage. The World Health Organization’s multi-country survey examined records from more than 300,000 deliveries in hospitals in 29 countries and found that coverage of key clinical interventions did not imply reduced mortality [5]. In India, a massive conditional cash transfer program dramatically increased facility delivery, but it appeared to have little effect on the MMR [6]. These and other studies raised alarm in global circles about the poor quality of clinical care in facilities, its deterrent effect on the utilization of facilities for childbirth, and its impact on maternal and newborn health [5,7–10].

Meanwhile, a parallel development in the human rights field was drawing attention to other aspects of quality in delivery care. Investigative reports by human rights organizations documented abusive and discriminatory treatment in labor and delivery rooms in Kenya [11] and the United States [12], in clear violation of human rights standards. This gave new urgency to an old phenomenon of routine childbirth that is medicalized and then managed in facilities in ways that undercut women’s efforts to maintain control over their birth experience, preserve their dignity, and safeguard their physical and emotional wellbeing [13–15]. Subsequent literature reviews described a range of disrespect and abuse during childbirth in health facilities, including nondignified care (e.g., shouting/scolding, threatening comments), neglect, lack of physical privacy, physical abuse, inappropriate demands for payment, and nonconsented care, and confirmed that this treatment is a worldwide phenomenon [16,17].

In the wake of these studies, civil society organizations have created the core of a newly energized global movement for respectful maternity care (RMC) [18]. The movement’s hundreds of members include organizations from high-, middle-, and low-income countries, representing patients, professional associations, academicians, activists, and other stakeholders. These parallel developments—in public health, human rights, and civil society advocacy—have created the foundation for action.

The *Staha* study (meaning “respect” in Swahili) was designed to build a conceptual and evidentiary basis to address disrespect and abuse in the United Republic of Tanzania and to inform the global RMC movement. In 2 districts of the Tanga Region, we conducted a baseline study to measure prevalence of disrespect and abuse. It found that approximately 20% of women reported at least 1 incident of disrespect and abuse during their delivery in these facilities [19]. Subsequent discussions with community members, health workers, and managers led to the design of a multicomponent intervention to reduce disrespect.

This paper reports on an intervention to reduce the prevalence of disrespect and abuse during childbirth in 2 districts of the Tanga Region of Tanzania.

## Methods

The study protocol was approved by the IRBs of Columbia University, Ifakara Health Institute, and the National Institute for Medical Research in Tanzania.

### The *Staha* intervention

**Fig 1:**
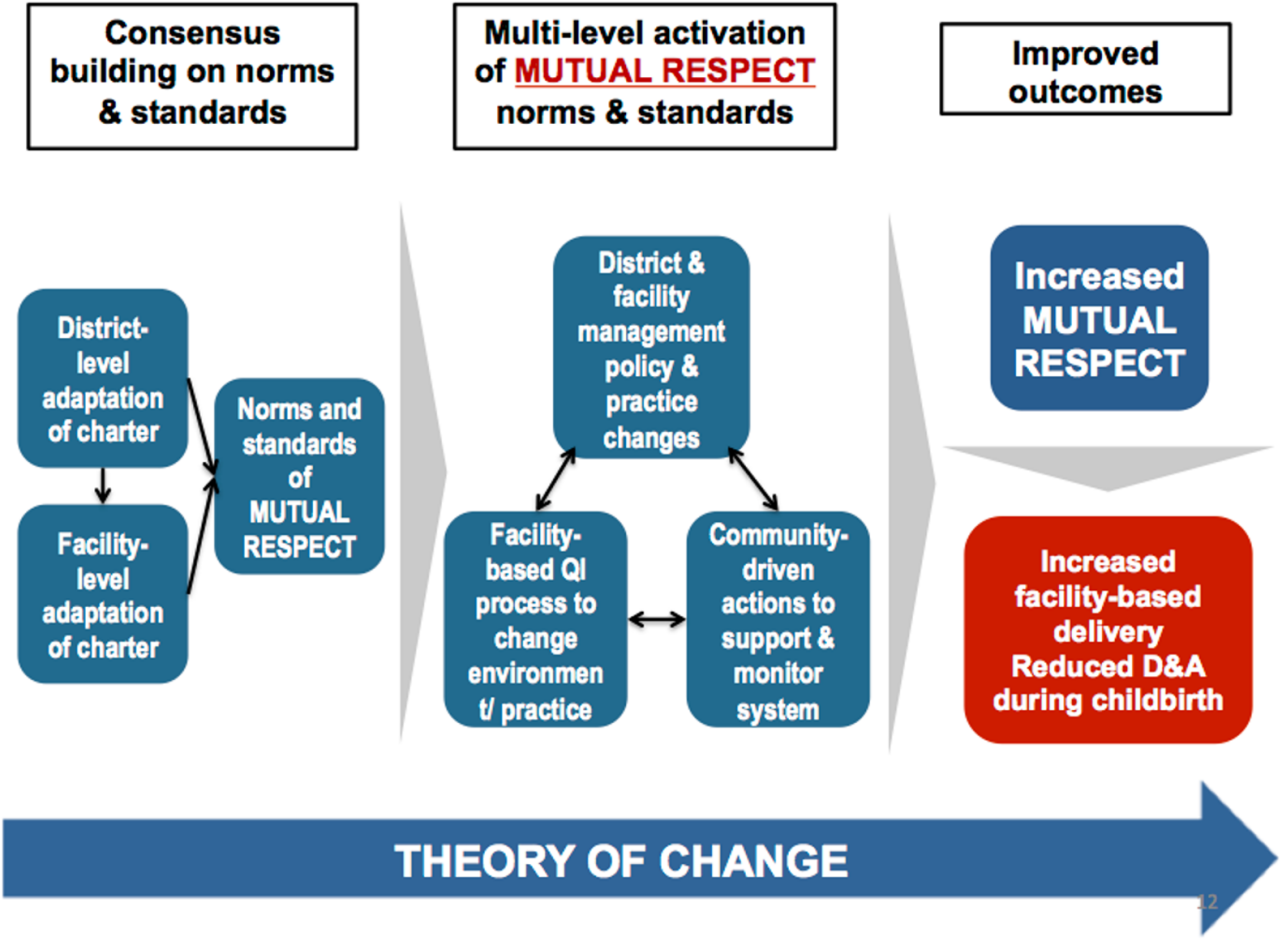
*Staha* project theory of change.

The intervention was developed through an iterative participatory process with local community and health system stakeholders that enabled them to analyze their own experience of disrespect and abuse in light of the baseline data and to consider potential actions to reduce it. Through this process, the *Staha* study identified a set of community and health system interventions that were intended to promote mutuality of respect between patients and providers. (See Fig 1 for the *Staha* intervention theory of change framework).

**Fig 2:**
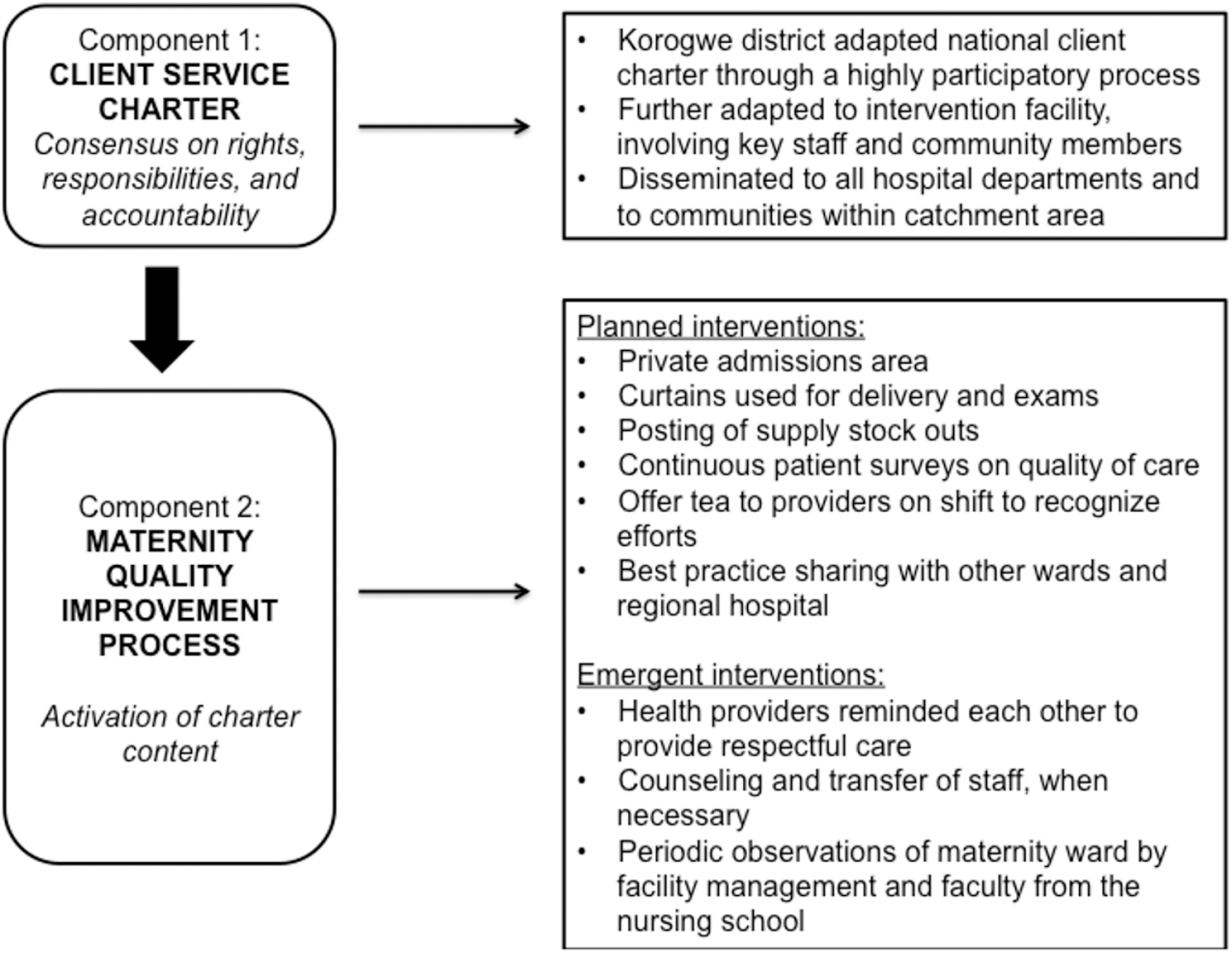
Description of *Staha* intervention components.

The intervention consisted of 2 main components (Fig 2). First, community and facility stakeholders together adapted a client service charter that had been promulgated by the Tanzanian government through the Ministry of Health and Social Welfare in 2005 but had lain dormant since that time. The local adaptation of the charter was drafted by a committee composed of the District Legal Officer, the Chairperson of the Council Health Service Board (CHSB), the Chairperson of the Social Welfare Committee of the District Council, the District Medical Officer, the District Hospital Medical Officer in-charge, the Chairperson of the District Hospital Governing Committee (HGC), the District Health Secretary, and a health center in-charge, as well as a village executive officer. This committee was selected based on recommendations from the district stakeholders and those involved in the participatory process. The adapted charter was then reviewed by 70 local stakeholders for feedback, including village executive officers, ward executive officers, district council authorities, health facility in-charges at intervention facilities, representatives from nongovernmental organizations (NGOs) in the district, and political leaders. Eighty-six percent of the stakeholders provided feedback. The main focus of the charter was to build consensus on norms and standards to foster mutual respect and respectful care. The charter was then disseminated to communities and posted in health facilities within the intervention district.

Second, the norms and standards articulated in the client charter were activated through a maternity ward quality-improvement process at 1 intervention facility, using tools from the Institute for Healthcare Improvement [20], to address disrespectful and abusive treatment as a system-level problem. Facilitated by members of our study team, maternity ward and hospital staff was presented with findings from the baseline and identified drivers of disrespect and abuse and proposed and prioritized interventions for change based on importance and feasibility. A quality-improvement team in the intervention hospital, consisting of staff from the maternity ward, reproductive and child health unit, pharmacy, and facility management, then facilitated the implementation of the interventions in the maternity ward and were responsible for tracking progress weekly. Maternity ward interventions were implemented 1 at a time and included moving the admissions area to a private room, using curtains for delivery and for physical examinations, posting supply stock outs to ensure transparency and build trust, and continuous customer satisfaction exit surveys anonymously filled by women who delivered in the ward. The latter were also used to monitor progress with the quality-improvement interventions and to decide when to implement the next intervention. The satisfaction surveys were analyzed by the quality-improvement team and discussed with the maternity ward staff. Staff in the maternity ward encouraged each other to treat women more respectfully. Quality-improvement interventions on the facility management level included tea provided to maternity ward staff, counseling of staff who continued to exhibit disrespectful behaviors, and best practice sharing with other wards and the regional hospital.

Although supported and facilitated by our study team, the implementation of the intervention was carried out by district, facility, and community stakeholders. The charter process took place over 6 months, after which our study team assisted in the facilitation of the quality-improvement process for 11 months. The intervention was then managed independently by facility managers and the regional quality improvement focal person for the following 10 months, at which point the endline survey commenced. Despite funding delays and turnover of key district and facility staff, the major components of the intervention were initiated within the intervention time frame and sustained beyond the endline survey.

### Study design and participants

We used a comparative before-and-after evaluation design to test the intervention to reduce disrespectful and abusive treatment of women during labor and delivery. As an implementation science study, this evaluation also collected qualitative and process data to identify potential mechanisms of change. Two districts in the Tanga Region of Tanzania, a rural region in the northeast corner of the country, were purposively chosen for the study. Tanzania has a maternal mortality ratio (MMR) of 398 per 100,000 live births [4], with 50.2% of deliveries occurring at health facilities [21]. The intervention was randomly assigned to Korogwe District with Muheza District as the comparison group. The intervention was measured at the facility level, with Korogwe District Hospital representing the intervention and Muheza District Hospital the comparison. The facilities are located approximately 60 kilometers apart.

At each facility, 2 surveys were performed, at baseline (December 2011 through May 2012) and 10 months after support for the intervention’s implementation ended (March through September 2015). Women aged 15 and over who delivered at the study facilities were eligible. Trained interviewers, unaffiliated with the facility, invited women discharged from the maternity ward to participate in an exit interview. All participants provided written informed consent. Interviews with eligible participants were conducted in tents outside of the maternity ward to ensure privacy. Women were given a bar of soap and a bottle of water in appreciation of their time and participation. Patients requiring support following disclosure of abuse were provided with a referral for mental health services at the regional hospital. The planned sample size for the study was 2,936 women: 734 women per district per time period (baseline and endline). Assuming the sample size, a 2-sided alpha of 0.05, and a 30% baseline prevalence of disrespect and abuse, there would be 80% power to detect a 15% decline in reported disrespect and abuse in the intervention facility compared to the comparison facility.

### Measures

The primary outcome of interest was self-reported experience of disrespectful or abusive treatment during labor and delivery. A woman was labeled as having experienced disrespect or abusive treatment if she answered “experienced” to any of the 14 questions about whether the specific disrespectful or abusive actions listed in Table 1 occurred during her labor and delivery. Secondary outcomes included affirmative responses for each of the questions in the categories of disrespect and abuse (Table 1). Individual questions and categories were based on a landscape analysis by Bowser and Hill and were further adapted and validated for the Tanzanian context with focus group discussions and in-depth interviews with recently delivered women and health system stakeholders [17]. We also explored the association between the intervention and delivery satisfaction and quality of care. Women were asked to rate their satisfaction with delivery, the respect providers showed them during delivery, and the quality of care during delivery. For satisfaction, responses were dichotomized from a 4-point Likert scale into very satisfied, somewhat satisfied, somewhat dissatisfied, or very dissatisfied. Quality measures were dichotomized into excellent, very good, fair, or poor.

**Table 1:** Disrespect and abuse categories and actions included in questionnaire and analysis.

Categories	Disrespect and abuse questions
**Nonconfidential care**	Patient's body seen by other people (aside from health provider) during delivery
** **	
**Nondignified care**	Health providers shouting at or scolding patient
** **	Health providers threatening to withhold treatment because patient could not pay or did not have supplies
** **	Health providers threatening patient for any reason or making negative or disparaging comments about the patient
** **	
**Neglect**	Health providers ignoring or abandoning patient when in need or when she called for help
** **	Delivered without any assistance
** **	
**Nonconsented care**	Tubal ligation (tying of fallopian tubes) without her permission
** **	Hysterectomy (getting your uterus removed) without patient or her relatives' permission
** **	Caesarean section without patient or her relatives' permission
** **	
**Physical abuse**	Hitting, slapping, pushing, pinching, or otherwise beating the patient
** **	Health providers sexually harassing patients or making sexual advances (for example, inappropriate touching or sexual comments that make you feel uncomfortable)
** **	Rape. By Rape I mean being forced to have intercourse or perform any other sexual acts against your will by someone other than your husband
** **	
**Inappropriate demands for payment**	Woman or baby not allowed to leave the hospital due to failure to pay
	Health providers suggesting or asking for a bribe or informal payment for better care

The literature suggests that a range of respondent and delivery experience factors are associated with report of disrespectful and abusive treatment during labor and delivery. Factors that were identified in past research included the following: respondent characteristics such as age, education, marital status, socioeconomic status, parity, reported low mood or depression in the last 12 months, and reported past physical abuse or rape [16,19,22,23]. Delivery experience factors included length of stay for delivery, Caesarean section, if the woman came directly to the facility for delivery, and any reported complications during childbirth. These same factors were included in a recent paper from the *Staha* study on prevalence and correlates of disrespect and abuse [19]. To measure socioeconomic status, we used a principal component analysis (PCA), developed by Filmer and Prichett, of 18 survey questions about household assets [19,24]. PCA index results were categorized into quintiles, with the lowest 2 quintiles (40%) classified as poor.

We assessed several process measures in the endline survey that related to the fidelity of the intervention, including questions on women’s experience regarding a range of respectful practices that providers were encouraged to adopt. Respectful maternity care questions were adapted from the Maternal and Child Health Integrated Program (MCHIP) Maternal and Newborn Quality of Care Survey [25].

### Statistical analyses

We first compared means and frequencies of baseline participant characteristics, including factors that may influence reporting of disrespect and abuse, by district using chi-square tests and *t* tests. Monthly baseline trends in reporting of disrespect and abuse were compared between intervention and comparison districts to confirm parallel trends, a key assumption of difference-in-difference analysis. This was done by regressing the main outcome on an interaction term between month of baseline data collection and district. Second, as per our prespecified analytic plan, we performed unadjusted logistic regression using a difference-in-difference approach for all primary and secondary outcomes using a dummy variable for facility, time (pre-post), and the interaction term of the 2 as a measure of the intervention impact. Finally, to test whether differences in women’s or facility characteristics influenced our estimates, we used a multivariable logistic regression to estimate a difference-in-difference model that included all variables in our conceptual model in addition to the above dummy variables. We followed the analysis plan as set forth in the IRB protocol except that we elected to adjust the final analysis for demographic variables to account for observed differences between the intervention and comparison group that might otherwise bias the association between the intervention and the outcome. For quality of care and satisfaction outcomes, we estimated relative risks using generalized linear models with a Poisson distribution, a log link, and robust standard errors to account for the high prevalence of these outcomes. Complete case analysis was used to permit comparability across models and avoid bias due to missing data. For the fidelity and process indicator measures, endline data from the 2 districts were compared using chi-square tests.

To address potential biases due to selection and contamination, sensitivity analyses were conducted by excluding participants who reported that they were aware of the intervention and by restricting analysis to those who lived in the nearby vicinity of the study facilities and thus were not drawn to the intervention hospital from the control catchment. All statistical analyses were performed with STATA (version 13). The trial is registered on the ISRCTN Registry (www.controlled-trials.com), number ISRCTN48258486.

## Results

At baseline, 2,085 women at the 2 study hospitals who had been discharged from the maternity ward after delivery were invited to participate in the survey. Of these, 1,388 (66.57%) agreed to participate. At endline, 1,680 women participated in the survey (72.29% of those approached). Women did not participate in the study largely due to the required wait time postdischarge for the administration of the interview. At baseline, there were some statistical differences between women delivering in the intervention hospital versus the comparison hospital (Table 2). A higher proportion of participants in the intervention facility than the comparison facility were married and of higher socioeconomic status, and a smaller proportion reported low mood or depression in the last 12 months or ever being physically abused or raped. Higher proportions of participants in the comparison facility had shorter lengths of stay for delivery and were more likely to come directly to the facility for delivery compared to women in the intervention facility. Other baseline characteristics were not statistically different. Preintervention trends in the main outcome between the 2 facilities did not differ significantly, with the exception of the first month of data collection, which was likely due to a small sample of surveys collected in that month.

**Table 2:** Baseline characteristics of survey respondents from 2 health facilities in Tanga Region, Tanzania, 2011–2012.

	INTERVENTION	COMPARISON	Difference	
	Baseline (*N* = 644)	Baseline (*N* = 744)		
	%	%	%	*p*-value
**Demographics**	** **	** **		
Age, mean	25.76	26.10	−0.34	(0.32)
Age categories				
15–19	16.77	13.31	3.46	(0.19)
20–34	71.58	74.06	−2.48	
35–50	11.65	12.63	−0.98	
Parity				
First birth	40.68	37.90	2.78	(0.30)
2–3 births	35.4	34.59	0.81	
4 or more births	23.91	27.46	−3.55	
Attended secondary education or greater	20.96	20.78	0.18	(0.93)
Married	84.47	79.27	5.20	(0.01)
Poor	35.07	41.22	−6.15	(0.02)
Household has electricity	28.93	22.37	6.56	(0.005)
Household has mobile phone	89.58	81.43	8.15	(0.00)
Reported low mood or depression in last 12 months	34.74	46.89	−12.15	(0.00)
Reported ever being physically abused or raped	2.65	12.79	−10.14	(0.00)
**Delivery care experience**				
Caesarean section	7.34	5.02	2.32	(0.07)
Reported any complications during childbirth^a^	56.54	55.15	1.39	(0.60)
Length of stay for delivery ≤1 day	34.32	24.45	9.87	(0.00)
Came directly to facility for childbirth	65.04	84.01	−18.97	(0.00)
**Maternity ward characteristics**				
Number of medical officers	1	2		
Number of annual deliveries	2827	3237		

^a^ Complications include extreme pain, high blood pressure, seizures, blurred vision, severe headaches, swelling in hands/feet, baby was in distress or too large, long labor (≥12 hours), excessive bleeding, and infection/fever.

Table 3 presents crude difference-in-difference estimates for all primary and secondary outcomes. There was a 3.39% (*p* < 0.0001) decrease in the percent of all women who experienced disrespect and abuse between the intervention and comparison facilities. Table 4 presents results from the multivariable logistic regression difference-in-difference analysis for the main outcome of interest. Complete data from baseline and endline were available for 2,983 women (97.23%) for this analysis. The intervention was associated with a 66% reduced odds (95% CI: 0.21–0.58, *p* < 0.0001) of a woman experiencing disrespect and abuse when adjusted for all covariates in the model. Women in the intervention facility were also significantly less likely to report events of neglect (OR: 0.36, 95% CI: 0.19–0.71, *p* = 0.045) and physical abuse (OR: 0.22, 95% CI: 0.05–0.97, *p* = 0.003) when adjusted for all variables in the conceptual model (Table 5). Finally, the intervention was associated with an increased likelihood of rating the respect providers showed them during their stay at the facility for delivery as excellent or very good (RR: 3.44, 95% CI: 2.45–4.84, *p* < 0.0001) and rating the overall quality of care for delivery as excellent or very good (RR: 6.19, 95% CI: 4.29–8.94, *p* < 0.0001) (Table 5). The process indicators show that women delivering in the intervention facility rated most elements of quality of care and respectful maternity care significantly higher than those in the comparison facility (Table 6).

**Table 3:** Unadjusted comparison of baseline to endline disrespect and abuse and process of care measures in 2 health facilities in Tanga Region, Tanzania.

	INTERVENTION DISTRICT				COMPARISON DISTRICT				Difference	
	Baseline (*N* = 644)		Endline (*N* = 1,001)		Baseline (*N* = 744)		Endline (*N* = 679)			
Primary outcome	*N*	%	*N*	%	*N*	%	*N*	%	%	*p*-value^a^
Any disrespect and abuse	84	13.10	32	3.20	163	22.27	107	15.76	−3.39	<0.0001
**Secondary outcomes**										
Disrespect and abuse score, mean (SD)	0.22	0.72	0.06	0.37	0.49	1.10	0.37	0.98	−0.04	0.55
Single-item disrespect and abuse	26	4.04	32	3.20	56	7.53	76	11.19	−4.50	0.04
Nonconfidential care	11	1.74	2	0.20	55	7.51	25	3.69	2.28	0.08
Nondignified care	35	5.46	22	2.20	110	15.03	76	11.19	0.58	0.06
Neglect	39	6.09	19	1.90	81	11.08	70	10.31	−3.42	0.001
Nonconsented care	0	0	0	0	1	0.14	0	0	0.14	–
Physical abuse	16	2.50	3	0.30	23	3.15	12	1.77	−0.82	0.03
Inappropriate demands for payment	15	2.37	0	0	5	0.68	4	0.59	−2.28	–
**Process of care measures**										
Very satisfied with delivery	552	85.98	924	92.31	515	70.26	511	75.37	1.22	0.98
Excellent/very good respect providers showed during delivery	66	10.28	177	17.68	207	28.24	116	17.11	18.53	<0.0001
Excellent/very good overall quality of care during delivery	51	7.94	279	27.87	171	23.33	102	15.04	28.22	<0.0001

^a^ *p*-value derived from logistic regression models; disrespect and abuse score is a summary score of the individual disrespect and abuse events listed in Table 1.

**Table 4:** Results from the difference-in-difference model of the association between intervention and reports of disrespect and abuse during childbirth (*N* = 2,983).

	OR	*p* value	95% CI	aOR	*p* value	95% CI
Difference-in-differences estimate (time period*district)	0.34	<0.0001	[0.21–0.57]	0.34	<0.0001	[0.21–0.58]
District	0.51	<0.0001	[0.38–0.69]	0.59	0.001	[0.43–0.81]
Time period	0.65	0.002	[0.49–0.85]	0.79	0.12	[0.59–1.06]
**Demographics**						
Ages 20–34 (reference group)						
Ages 15–19				1.19	0.32	[0.84–1.68]
Ages 35–48				1.19	0.40	[0.79–1.81]
Attended secondary education or greater				1.25	0.13	[0.93–1.68]
Married				0.83	0.23	[0.61–1.13]
2–3 births (reference group)						
First birth				1.44	0.02	[1.06–1.96]
4 or more births				1.18	0.38	[0.82–1.70]
Poor				1.17	0.22	[0.91–1.49]
Reported low mood or depression in last 12 months				1.25	0.07	[0.98–1.59]
Reported ever being physically abused or raped				2.33	<0.0001	[1.60–3.40]
**Delivery experience**						
Length of stay for delivery ≤1 day				1.33	0.03	[1.03–1.73]
Caesarean section				0.98	0.93	[0.55–1.73]
Reported any complications during childbirth				1.94	<0.0001	[1.52–2.48]
Came directly to the facility for childbirth				0.69	0.009	[0.53–0.91]

aOR, adjusted odds ratio; OR, odds ratio

**Table 5:** Results from the difference-in-difference model of the association between the intervention and reports of secondary outcomes (*N* = 2,983).

	aOR^a^	*p* value	95% CI
**Disrespect and abuse outcomes**			
Nonconfidential care	0.25	0.09	[0.05–1.23]
Nondignified care	0.58	0.10	[0.30–1.12]
Neglect	0.36	0.003	[0.19–0.71]
Physical abuse	0.22	0.045	[0.05–0.97]
**Satisfaction and quality of care**^b^			
Very satisfied with delivery	0.98	0.67	[0.91–1.06]
Excellent/very good respect providers showed for delivery	3.44	<0.0001	[2.45–4.84]
Excellent/very good overall quality of care for delivery	6.19	<0.0001	[4.29–8.94]

^a^ Models adjusted for district, time period, age, education, marital status, parity, poor, reported low mood or depression in last 12 months, reported ever being physically abused, length of stay for delivery ≤1 day, Caesarean section, reported any complications during childbirth, came directly to the facility for childbirth

^b^ adjusted relative risks

aOR, adjusted odds ratio

**Table 6:** Implementation fidelity: Respectful care process indicators at endline, 2015.

	INTERVENTION (*N* = 1,001)		COMPARISON (*N* = 679)		chi-sq *p*-value
	*N*	%	*N*	%	
Providers came quickly when called for them	892	89.56	548	80.95	<0.0001
Respectfully greeted you when you arrived at the maternity ward	572	58.73	512	75.41	<0.0001
Asked if you had any questions during your stay in the maternity ward	686	68.88	253	37.32	<0.0001
Supported you during labor in a friendly way	810	81.00	492	72.46	<0.0001
Encouraged you to walk or assume different positions during labor	761	76.10	366	53.90	<0.0001
Encouraged you to consume liquids/food throughout labor	715	71.50	461	67.89	0.11
Assisted you to use toilet facilities	257	25.70	185	27.25	0.48
Able to communicate with your relatives or other persons who accompanied you	274	27.37	67	9.88	<0.0001
Received any assistance to reduce pain	124	12.39	22	3.24	<0.0001
Excellent/very good language providers used towards patients	119	11.89	36	5.30	<0.0001
Excellent/very good physical privacy during delivery	393	39.30	190	27.98	<0.0001
Excellent/very good information provided about availability of drugs and supplies	118	11.79	9	1.33	<0.0001
Excellent/very good cleanliness of the facility	55	5.50	3	0.44	<0.0001
Rating of this delivery compared to previous delivery at the same facility (*N* = 565)					
Better	342	85.93	132	43.28	<0.0001
Same	54	13.57	159	52.13	
Worse	2	0.50	14	4.59	

## Discussion

This study found that after a participatory community-health system intervention in Tanga Region, Tanzania, the likelihood of self-reported disrespectful and abusive care during labor and delivery was significantly reduced (66% reduced odds). The largest reduction was for physical abuse, followed by neglect. Process indicators showing better patient-reported quality of care in the intervention facility, including respectful treatment from providers, support the likelihood that the intervention was responsible for the reduction in disrespect and abuse. Importantly, these effects were still observed nearly one year after the end of *Staha*’s facilitation of implementation in the intervention district, indicating the potential for sustainability.

While the size of the absolute reduction in disrespectful treatment in the intervention facility was large (from 13.10% to 3.20%), there were reductions in the comparison facility as well (from 21.27% to 15.76%, Table 3). Although this represents a substantial reduction in the risk of experiencing disrespect and abuse for an individual woman, there was a relatively small difference between the intervention and comparison facilities in prevalence reduction (3.39%). This was likely due to quality changes that occurred in the comparison facility over the study period, including the posting of patients’ rights in the maternity ward and a pharmacy price list, and delivery room renovations to include cubicles for privacy.

There have been several other efforts to design, implement, and assess interventions specifically aimed at reducing disrespect and abuse. In Kenya, where the early human rights reports had garnered significant public attention, the *Heshima* project used multiple interventions, including maternity open days for prospective patients to visit the facility, values-clarification workshops for providers, and dispute-resolution training for community leaders. They reported a 7% reduction in the proportion of women reporting disrespect and abuse [26]. However, the study did not include a comparison group and coincided with broader health system reforms that may have affected the results. In Tanzania, an intervention conducted in a high-volume referral hospital in Dar es Salaam used maternity open days to improve patient knowledge and awareness, combined with a respectful care workshop to sensitize and empower providers. These interventions, developed through a participatory process [27], were shown to have a positive effect on multiple proximate indicators, including patient knowledge of rights and birth preparedness, provider attitudes, and patient-provider communications [28]. However, the study was not designed or powered to measure impact on prevalence of disrespect and abuse, nor was a comparison group included. To our knowledge, the *Staha* study reported here is the first comparative before-and-after evaluation of an intervention to address disrespect and abuse during childbirth that includes a comparison group to reduce the likelihood that secular trends or other factors that could explain the observed change.

In *Staha*, as in the few other projects that have tried to tackle disrespect and abuse explicitly, we adapted tools from several related fields, including quality improvement, social accountability, and behavior change. Each of these tools and techniques has a mixed record in the literature [29,30]. Systematic reviews generally show that their effect depends on whether, in context, their implementation activates deeper change mechanisms [31,32]. Thus, the mechanism of action is never the tool itself but the entire process by which the problem is identified and analyzed, the intervention chosen, its use negotiated and practiced, and its effects assessed and understood [33]. And this implementation process likely works in a sustained way only when it engages the organizational culture and power dynamics at the heart of disrespect and abuse [34–36].

This study had some limitations. First, we found several differences between the characteristics of women in the 2 groups at baseline. We controlled for these factors in our regression analyses, and our finding of parallel trends for reports of disrespect and abuse in the pretreatment data supported the notion that the comparison district was a plausible counterfactual for the intervention district. Second, the response rate for both baseline and endline introduces the possibility of selection bias. However, women in our sample were comparable in terms of age and parity to the larger delivery population in the facilities, providing confidence that participants are representative of the sample population. Our sample had a lower proportion of women who had Caesarean sections than the facility delivery population. In previous study analyses, having a Caesarean section was not associated with self-report of disrespect and abuse upon discharge after childbirth [19]. Third, it is possible that women may have self-selected into the intervention facility or that there was contamination from the comparison district, due to exposure to the client charter and/or word of mouth about the quality-improvement intervention. These women may have had a more positive expectation and subsequently more positive reporting of their care. However, elimination of women who reported that they had heard of the intervention did not alter our findings (S1 Table), nor did restricting our sample to participants who lived in the nearby vicinity of the hospitals and were therefore not likely to be bypassing other facilities. Fourth, it is possible that other unmeasured changes in the facilities during the study period, such as turnover in staff or facility management, may have influenced study results. We did not identify any such changes that were major enough to explain the large effect we saw. Fifth, even though we observed a smaller baseline prevalence and reduction in disrespect and abuse than assumed in the power calculation, because we still found a significant result, our study was appropriately powered. Sixth, we used logistic regression in instances in which we believe the odds ratio was a good approximation of the risk ratio due to the rare disease assumption. In the instances in which the outcome was common (i.e., >20%), we opted to use relative risk regression to obtain our estimates of interest. Lastly, this is a proof-of-concept study, as only 2 hospitals in Tanzania were included. Future, larger-scale research should be undertaken to evaluate the applicability of this approach to other settings.

Our findings have important implications moving forward as the RMC field evolves. It is tempting in global- and national-level discussions to conceptualize the next challenge as the translation of global standards of respectful care into practice by identifying the most-effective intervention through studies such as *Staha*. However, insights from implementation science, behavioral science, and organizational science would all warn against a simple translational approach [37]. Eliminating disrespect and abuse requires individual behavior change, organizational and systems change, and, ultimately, deeper societal transformation. These are complex, multidimensional challenges that do not evaporate just by order of a court or mandate of a minister [38]. There will never be a simple, single technical fix to identify and prescribe. Thus, unlike with a new drug or biomedical procedure, our goal in testing these interventions to mitigate disrespect and abuse should not be to assert definitively which tool works best, so that it can be mandated, funded, and promoted widely. Instead, initiatives such as *Staha* contribute to the field by demonstrating promising strategies for enabling and supporting frontline community- and facility-based actors to identify, confront, and act on both the symptoms and causes of disrespect and abuse in their own settings.

## Conclusion

This study provides evidence that a participatory community-health system intervention that articulates new norms, standards, and practices for mutual respect between patients and providers and supports their implementation through facility-based management and health provider reflection is a potential strategy to reduce the prevalence of disrespect and abuse during childbirth. The magnitude of the effects observed here suggests that this is a promising direction for future efforts to reduce disrespect and abuse. Future initiatives to build on the *Staha* findings should carefully adapt the intervention to local context, retain the active participation of key stakeholders, and explore efficient means for scaling it both geographically and institutionally by identifying the particular changes needed at higher levels of the health system to sustain such practices at the frontline [39,40].

Finally, improvements in technical quality of care, and in human resource and commodities availability, should accompany efforts to humanize care to address persistently high rates of maternal and newborn deaths in health facilities in many low-resource settings. Developing quality-improvement strategies that can tackle both clinical competence and compassion, supported through community accountability mechanisms, should be a global priority.

## Supporting information

S1 Table Results from difference-in-difference model of the association between the intervention and reports of disrespect and abuse during childbirth, excluding participants who had heard of the intervention (*N* = 2701). (PDF)

S1 Text STROBE checklist. (DOC)

S2 Text Analytic plan from IRB protocol. (DOC)

## Abbreviations

aOR: adjusted odds ratio
MDG: Millennium Development Goal
MMR: maternal mortality ratio
NGO: nongovernmental organizations
OR: odds ratio
PCA: principal component analysis
RMC: respectful maternity care
