# Supplementary material for: Community and health system intervention to reduce disrespect and abuse during childbirth in Tanga Region, Tanzania: A comparative before-and-after study
Source: PLoS Med. 2017 Jul 11;14(7):e1002341. doi: 10.1371/journal.pmed.1002341 (PMC5507413; doi:10.1371/journal.pmed.1002341)
Supplement: S1 Table — (PDF) [file pmed.1002341.s001.pdf]

	OR		95% CI
Difference in differences estimate (time period*district)	0.35	***	[0.20 - 0.62]
**Demographics**			
Ages 20-34 (reference group)			
Ages 15-19	1.21		[0.85 - 1.72]
Ages 35-48	1.20		[0.78 - 1.84]
Attended secondary education or greater	1.34		[0.99 - 1.81]
Married	0.83		[0.61 - 1.13]
2-3 births (reference group)			
1st birth	1.37		[1.00 - 1.88]
4 or more births	1.12		[0.77 - 1.63]
Poor	1.17		[0.91 - 1.50]
Reported low mood or depression in last 12 months	1.27		[0.99 - 1.62]
Reported ever being physically abused or raped	2.27	***	[1.54 - 3.34]
**Delivery experience**			
Length of stay for delivery ≤ 1 day	1.36	*	[1.05 - 1.77]
Caesarean section	1.00		[0.56 - 1.78]
Reported any complications during childbirth	2.05	***	[1.59 - 2.63]
Came directly to the facility for childbirth	0.72	*	[0.54 - 0.95]

* $p \leq 0.05$, ** $p \leq 0.01$, *** $p \leq 0.001$; all models controlled for district and time period
