# Supplementary material for: Community and health system intervention to reduce disrespect and abuse during childbirth in Tanga Region, Tanzania: A comparative before-and-after study
Source: PLoS Med. 2017 Jul 11;14(7):e1002341. doi: 10.1371/journal.pmed.1002341 (PMC5507413; doi:10.1371/journal.pmed.1002341)
Supplement: S2 Text — (DOC) [file pmed.1002341.s003.doc]

**IRB PROTOCOL – ANALYTIC PLAN**

**Promoting Respectful and Attentive Care in Tanzania**

8 September 2011

Study Design and Sampling:

*Effectiveness evaluation:* We will evaluate the effects of the program activities on reported level of abuse and facility delivery rates. This will be done in a quasi-random, pre-post comparison group study as per below:

O X O (program district)

O O (comparison district)

The districts have been selected to be similar in population demographics, facility number and type, and delivery volumes. These two districts also do not have any ongoing maternal health programs that could confound findings.

The X refers to the multi-pronged program that will derive from the baseline assessment. The O’s refer to observations—in this case, data on prevalence of abuse from exit surveys and facility data on number of facility deliveries. The program activities are scheduled to be implemented for 12 months. Four months of data will be collected at baseline and four after program activity implementation has concluded.

This aim of this component of the research is to assess two types of outcomes:

• ***Increases in utilization***. These will be assessed using a difference-in-difference regression analysis comparing the difference in mean de-seasoned monthly visits before and after the program activities in program facilities to the same measure in comparison facilities. Our previous work in Kigoma Region, Tanzania found that of rural women who delivered their last child at home, 8.8% reported poor provider attitude, 3.8% reported they tried to get but were denied care, and 3.0% reported being treated badly in the past as reasons for not using a facility (women could select multiple answers; unpublished data from population-based study). This is based on a rural sample of women, many of whom live far from hospitals and health centers that they consider adequate for delivery. The importance of poor attitude as a deterrent for utilization may be proportionally higher for women living closer to facilities who are not facing major transportation barriers.

• ***Reductions in reported abuse/disrespect***. The facility exit survey used in the baseline assessment will be used to collect data on prevalence of abuse before and after program activity implementation in the program district and simultaneously in the comparison district. Reductions in abuse will be assessed using t-tests to compare overall levels and subtypes of abuse pre and post the program activities in program district facilities. This will be compared to changes in reported abuse in comparison district facilities to account for potential secular (maturation) and history threats (e.g., other region-wide changes).

**3. STUDY PROCEDURES and 5. STUDY INSTRUMENTS**

Facility exit questionnaires will utilize a structured, in-person questionnaire. A copy of this draft instrument is attached to this protocol (Appendix B). This questionnaire will take approximately 45 minutes to complete. The questionnaire is comprised of eight modules: Demographics, Household Characteristics, Asset Index, Past Service Utilization, Delivery Characteristics, Perceived Quality and Satisfaction, Experience of Disrespect and Abuse, and Looking Forward. This questionnaire—and particularly the section on Experience of Disrespect and Abuse—will be modified following qualitative work.

Objective 5 (effectiveness evaluation) evaluates the effects of the program activities using a facility exit survey and measurement of facility deliveries.

The facility exit survey will use the same instrument as used in the baseline assessment and above.

Measurement of facility deliveries will utilize facility monthly reports. This instrument contains measures of normal vaginal deliveries, instrumental deliveries, caesarean sections, ante-/intra-/post-partum referrals, and facility case fatalities.
